# 

**Published:** 1894-05-19

**Authors:** 


CONTENTS.

Rational Therapeutics and Therapeuticians - 133
Around the Hospitals - 134
On Modern Progress in Ophthalmic Medicine and Surgery. By ROBERT BRUDENELL CARTER, F.R.C.S. - 135
The First Century of English Medical Literature.—III. The Library of the Physician - 136
The Localisation of Sensation in the Brain - 137
The Field of Science - 138
Annotations.—Paranoiacs—An Unexpected Cure—The Lister Portrait - 139
Notes and News - 140
Scraps and Gleanings - 152
Medical Progress and Hospital Clinics—
Tuberculosis - 141
Intra-Uterine Syringing in Puerperal Septicæmia - 142
MEDICAL PROGRESS—Progress in Dermatology - 143
PROGRESS IN SURGERY.—Cranial Surgery - 146
NEW APPLIANCES AND THINGS MEDICAL - 147
The Institutional Workshop—
HOSPITAL ADMINISTRATION - 146
THE STORY OF THE INSANE FROM YEAR TO YEAR - 150
HOSPITAL FESTIVALS AND MEETINGS - 151
CONSTRUCTION NOTES - 152
The Book World of Medicine and Science - 153

EXTRA SUPPLEMENT.—THE NURSING MIRROR.

News from the Nursing World.—Nurses and Vaccination Guardians and Nurses—Duty First—Asylum Patients—A Small Return—A New Nurses' Home—District Nurses at Stratford—District Nursing at Lewes—Enthusiasm at Kettering—Unique Methods—Nursing in Glasgow—Lectures in Melbourne—San Francisco Nurses—Short Items - lxi

On General Nursing. XII.—Pneumonia (*continued*) - lxiii

A Country Union - lxiii

Women's Work.—Choice of a Profession - lxiv

Novelties for Nurses - lxiv

Ladies in Australia - lxiv

Nursing in the West Indies - lxv

Acqui and Its Mud Baths - lxv

Where to Go - lxvi

Thoughts on Training - lxvi

Our American Letter - lxvii

Presentations—Appointments - lxvii

A Visit to Canada. I.—The Prairie - lxviii

Notes and Queries - lxviii

The Muses' Looking-Glass. The Defence of Cosmetics - lxix

Everybody's Opinion - lxx

The Medico-Psychological Association - lxx

Minor Appointments - lxx

For Reading to the Sick - lxx

